# Acetabular cup fixation with and without screws following primary total hip arthroplasty: migration evaluated by radiostereometric analysis

**DOI:** 10.1177/11207000231164711

**Published:** 2023-04-05

**Authors:** Jennifer S Polus, Edward M Vasarhelyi, Brent A Lanting, Matthew G Teeter

**Affiliations:** 1 School of Biomedical Engineering, Western University, London, Ontario, Canada; 2 Imaging Research Laboratories, Robarts Research Institute, Western University, London, Ontario, Canada; 3 School of Biomedical Engineering, Collaborative Specialization in Musculoskeletal Health Research, and Bone and Joint Institute, Western University, Canada; 4 Division of Orthopaedic Surgery, Schulich School of Medicine and Dentistry, Western University, London, Ontario, Canada; 5 Department of Medical Biophysics, Schulich School of Medicine and Dentistry, Western University, London, Ontario, Canada

**Keywords:** Acetabular cup, migration, radiostereometric analysis, screw fixation, total hip arthroplasty

## Abstract

**Background:**

Early cup migration after total hip arthroplasty (THA) is correlated to late revision due to aseptic loosening. However, the use of screws for increased cup stability remains unclear and debated. The purpose of this study is to assess acetabular migration between cups fixated with and without the use of screws.

**Methods:**

Patients underwent primary THA using either a direct anterior (DA) or a direct lateral (DL) approach. The DA surgeon routinely supplemented cup fixation with 1 or 2 screws while the DL surgeon used no screws. At 7 follow-up visits up to 2 years post operation, patients underwent radiostereometric analysis (RSA) imaging for implant migration tracking. The primary outcome was defined as proximal cup migration measured with model-based RSA.

**Results:**

68 patients were assessed up to 2 years post operation, *n* = 43 received screws and *n* = 25 did not. The use of screws had a significant effect on cup migration (*p* = 0.018). From 2 weeks to 2 years post operation, the total mean migration was 0.403 ± 0.681 mm and 0.129 ± 0.272 mm (*p* = 0.319) for cups with and without screws, respectively. The number of screws used also had a significant impact, with cups fixated with 1 screw migrating more than cups fixated with 2 (*p* = 0.013, mean difference 0.712 mm).

**Conclusions:**

Acetabular cups fixated with only 1 screw resulted in greater migration than cups with no screws or 2 screws, though the mean magnitude was well under the 1.0 mm threshold for unacceptable migration. However, 3 of the 24 patients who received only 1 screw exceeded the 1.0 mm threshold for unacceptable migration. Ultimately, the results of this study show that the use of 2 screws to supplement cup fixation can provide good implant stability that is equivalent to a secure press-fit component with no screws.

Clinical trial registration: ClinicalTrials.gov (NCT03558217)

## Introduction

Total hip arthroplasty (THA) is the only viable treatment for end-stage hip osteoarthritis, with high long-term survivorship and success for most patients. Still, with the continuous increase in number of THA procedures performed, comes an inevitable parallel increase in the number of revision THA procedures, especially due to the increased rate of THA in younger patients.^1,2^ Aseptic loosening of implant components is 1 of the main causes for revision THA and results in substantial clinical and economic burdens.^3–5^

Early migration of the acetabular cup can lead to aseptic loosening and has been reported to be correlated to late revision.^6–8^ Thus, initial stability of the acetabular component is essential to improve longevity and decrease the risk of revision. The use of screws in uncemented THA is intended to support and aid in the primary fixation of the press-fit cup; however, whether this is the case remains an unclear and debated topic in literature.^9,10^ A meta-analysis performed by Fei et al.^11^ found screws had no significant impact on cup stability, however, a study by Tabata et al.^12^ indicated increased primary stability of the cup with the use of screws. Further, a registry study found that screws did not provide protection against acetabular cup loosening but also did not have any long-term negative consequences.^13^ As such, the use of screws to fixate the acetabulum cup is often left to surgeon preference and discretion.

Radiostereometric analysis (RSA) is recognised as the gold standard for implant migration tracking, with an accuracy of 0.2 mm of translation and 0.5 degrees for rotations.^14,15^ RSA plays an important role in evaluating implant fixation following THA and can be used to compare implant performance between patient groups. Pijls et al.^7^ defined thresholds of proximal cup migration deemed to be acceptable, at risk for higher revision rates, and unacceptable as <0.2 mm, between 0.2 mm and 1.0 mm, and >1.0 mm, respectively. With these well-defined thresholds in mind, the purpose of the present study was to use model-based RSA to evaluate acetabular cup migration following THA between acetabular cups fixated with and without the use of screws.

## Material and methods

### Study design and participants

The present study is a secondary analysis of a prospective clinical trial that was approved by our institutional research ethics board and registered with ClinicalTrials.gov (NCT03558217). Patients undergoing unilateral primary THA were eligible to participate in the initial prospective randomized control trial (RCT).^16^ Patients were prescreened and excluded based on the following criteria: symptomatic contralateral hip OA, revision or bilateral THA, a body mass index (BMI) >40 kg/m^2^, cognitive defects or neuromuscular disorders that would prevent a walking test, inability to understand English, and if the patient lived >100 km from our institution due to frequent follow-up visits. Eligible patients were recruited and provided written informed consent prior to participation.

Depending on their surgeon referral, patients recruited to this study underwent THA using either the direct lateral (DL) or the direct anterior (DA) surgical approach. 2 fellowship-trained arthroplasty surgeons specialised in their respective approach performed all the operations; 1 surgeon (EMV) performed all the DL surgeries, and 1 surgeon (BAL) performed all the DA surgeries. Although surgical approach was not randomised, the expertise-based study design used has been reported to reduce challenges related to differential expertise bias and clinical equipoise in orthopaedics.^17,18^ All patients received a cementless Pinnacle cup with AltrX highly crosslinked acetabular liner (DePuy Synthes, Warsaw, IN, USA), a cobalt-chromium head, and a collared or collarless cementless Corail femoral stem. Both surgeons preoperatively templated to determine the final cup size for each patient. The DL surgeon began cup implantation by reaming 3 sizes under the templated cup size and increased in odd size reamers, with the final ream being 1 mm under the desired cup size. The DA surgeon began by reaming with a 44-mm reamer to get the appropriate depth of the cup, and then also chose a reamer 1 mm below the templated cup size.

The DA surgeon routinely opts to supplement acetabular press-fit cup fixation with the use of 1 or 2 screws as his standard surgical procedure, while the DL surgeon uses no screws as their routine. For screw placement, the DA surgeon affirmed the appropriate screw hole position as part of the multi-screw hole cup insertion and after suitable visualisation, each hole was drilled under direct visualisation with the appropriate drill bit and guide. As needed, the depth gauge was used to confirm depth and containment within bone. The appropriate screw length was then selected for each hole and inserted with the applicable screwdriver. Patients received either 1 or 2 screws in accordance with surgeon discretion based on adequacy of bone stock and purchase of the first screw at the time of screw insertion. For acetabular cup placement, the DL surgeon targets an inclination of 40–45° and an anteversion that is parallel to the transverse acetabular ligament. The DA surgeon targets an inclination of 30–35° and an anteversion of 10–20°. Although each surgeon has a different cup placement target, both fall into the recommended safe zones.^19,20^ To enable implant migration tracking with RSA, a minimum of 3 1-mm diameter tantalum beads were inserted into the pelvic bone surrounding the cup intraoperatively.

### Radiographic analysis

All patients underwent RSA imaging in a supine position at 2, 4, and 6 weeks, 3 and 6 months, and 1 and 2 years postoperatively. A uniplanar calibration cage (RSA Biomedical, Umea, Sweden) was used to define the coordinate system and ensure patients were consistently positioned at each exam. Model-based RSA software (RSAcore, Leiden, The Netherlands) was used to analyse the acquired radiographs. Positive translation directions were defined as proximal translation in the y-axis, medial translation in the x-axis, and anterior translation in the z-axis. Positive rotation directions were defined as internal rotation about the y-axis, anterior rotation about the x-axis, and adduction about the z-axis.^21^

To further evaluate acetabular cup position, anteversion and inclination angles were measured from the anteroposterior (AP) and lateral radiographs acquired at the standard 6-week postoperative visit. The anteversion angle was measured on the lateral radiograph using the ischiolateral method, where the ischium was used as an anatomical landmark. The anteversion angle was defined as the angle between a reference line made perpendicular to the long axis of the ischium and a line across the opening of the cup minus 26°.^22,23^ The inclination angle was measured from the AP radiograph, defined as the angle between the bi-ischial line and a line tangent to the opening of the cup.^23^

### Additional outcome measures

Patient demographic information was obtained from the hospital’s database. Patients also completed the Short-Form 12 (SF-12), Western Ontario and McMaster Universities Osteoarthritis Index (WOMAC) and University of California Los Angeles (UCLA) Activity Score questionnaires preoperatively, and at 3 months, and 1 and 2 years postoperatively. Clinicians also completed the Harris Hip Score (HHS) evaluation for each patient at these visits.

### Statistical analysis

All statistics were completed using Prism version 9.3.1 (GraphPad Software) and data were reported as mean and standard deviations. Unpaired *t*-tests were used to compare age, BMI, questionnaire scores, and anteversion and inclination angles between patient groups and a Fishers exact test was used to detect a difference in sex and cup size. A mixed-effects model with Sidak’s multiple comparison tests was used to compare acetabular cup migration between patient groups over time. Statistical significance was set at a *p*-value ⩽ 0.05.

The presented work is a secondary analysis of a prospective study that was designed to detect changes in femoral stem subsidence between stem designs.^16^ As such, the sample size of the study was not designed to be powered to detect differences in acetabular cup migration. A post-hoc computation of achieved power, given an alpha of 0.05, the number of subjects, and an effect size of 0.27, calculated based on the 2-year proximal migration measurements revealed the achieved power was 83%.

## Results

**Figure 1.**
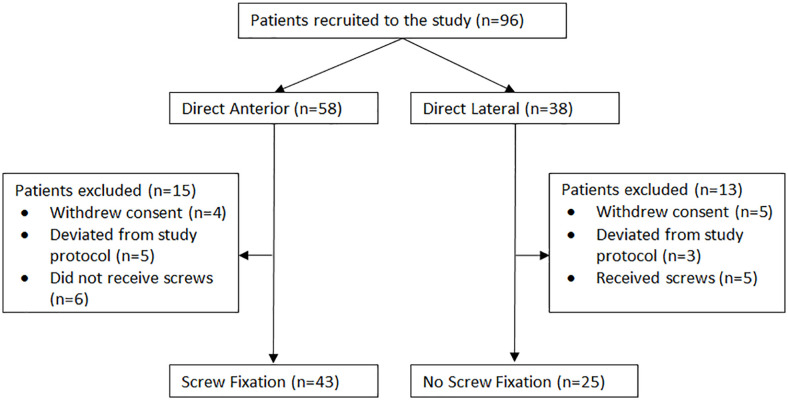
CONSORT study flow diagram.

A total of 96 patients consented to participate in the initial prospective study,^16^ whereby 58 underwent the DA approach and 38 underwent the DL approach. 28 patients were excluded from the study, where 9 patients withdrew their consent and 8 patients who deviated from the initial study protocol by receiving a different femoral stem design or not receiving any RSA beads needed for implant migration tracking. Further, 11 patients were excluded from the analysis for deviating from their screw fixation group. There were a total of 68 patients analyzed in the presented study, whereby 43 underwent the DA approach and received screws and 25 underwent the DL approach and did not receive screws (Figure 1).

There were no differences in demographics (Table 1) or questionnaire scores (Supplemental Table 1) between patient groups. There was a significant difference in acetabular cup position measured radiographically between groups (Supplemental Figure 1). The mean inclination angle for the screw fixation group and the no screw fixation group was 32.64° and 41.71°, respectively (mean difference = 9.07°, *p* < 0.0001). The mean anteversion angle for the screw fixation group and the no screw fixation group was 21.32° and 14.90°, respectively (mean difference = 6.42°, *p* = 0.0499). No patients required any revision surgery for their acetabular cup.

**Table 1.** Demographics and radiological analysis between patient groups. Values are the actual numbers or reported as mean ± standard deviation.

Characteristic	Screw fixation	No screw fixation	*p*-Value
Sex *(F:M)*	18:25	13:12	0.458
BMI *(kg/m*^2^*)*	29.26 ± 4.85	27.53 ± 4.81	0.159
Age *(years)*	65.40 ± 8.03	65.44 ± 9.88	0.984
Acetabulum cup diameter	2 of 48 mm 4 of 50 mm 11 of 52 mm 6 of 54 mm 8 of 56 mm 7 of 58 mm 1 of 60 mm 4 of 62 mm	1 of 48 mm 6 of 52 mm 5 of 54 mm 5 of 56 mm 3 of 58 mm 4 of 60 mm 1 of 62 mm	0.374
Inclination angle	32.64° ± 4.88°	41.71° ± 5.71°	<0.0001
Anteversion angle	21.32° ± 11.88°	14.90° ± 12.52°	0.0499

BMI, body mass index.

**Figure 2.**
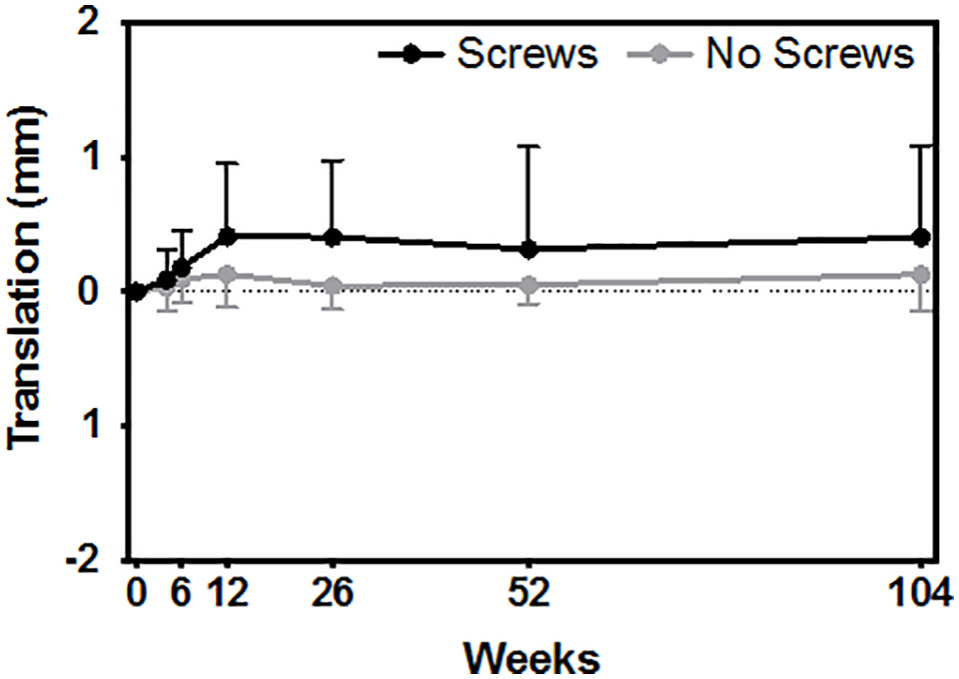
Comparing proximal migration (y-axis translation) between cups fixated with or without screws (*p* = 0.018).

The use of screws to supplement cup fixation had a significant effect on the proximal migration of the acetabulum cups (Figure 2) (Table 2) *p* = 0.018. At 6 months post operation, the screw fixation group migrated significantly more than the group with no screw fixation (mean difference = 0.360 mm, *p* = 0.015). From 2 weeks to 2 years post operation, the mean proximal migration was 0.403 ± 0.681 mm and 0.129 ± 0.272 mm for the cups with and without screws, respectively (*p* = 0.319). There were no other differences in the other axes of translation or rotation between screw fixation groups.

**Table 2.** Translations and rotations of the acetabulum cup from the 2-week baseline exam between screw and no screw fixation groups. Values are reported as mean ± standard deviation.

Plane	Screw fixation						No screw fixation						*p*-Value
	4 Weeks	6 Weeks	12 Weeks	26 Weeks	52 Weeks	104 Weeks	4 Weeks	6 Weeks	12 Weeks	26 Weeks	52 Weeks	104 Weeks	
Translation (mm)													
x	0.02 ± 0.37	0.03 ± 0.59	-0.09 ± 0.88	-0.21 ± 0.88	-0.13 ± 1.02	-0.37 ± 1.09	-0.01 ± 0.37	0.02 ± 0.22	0.03 ± 0.35	-0.10 ± 0.39	0.03 ± 0.23	-0.05 ± 0.39	0.499
y	0.09 ± 0.23	0.18 ± 0.28	0.41 ± 0.54	0.41 ± 0.57	0.32 ± 0.76	0.40 ± 0.68	0.03 ± 0.18	0.09 ± 0.17	0.13 ± 0.25	0.05 ± 0.18	0.05 ± 0.14	0.13 ± 0.27	0.018
z	-0.08 ± 0.93	-0.19 ± 1.23	-0.33 ± 1.12	0.11 ± 0.92	-0.01 ± 1.18	0.22 ± 1.06	0.04 ± 0.32	-0.04 ± 0.38	-0.10 ± 0.42	-0.05 ± 0.44	0.03 ± 0.33	0.05 ± 0.53	0.900
Rotation (degrees)													
x	-0.12 ± 1.14	-0.42 ± 1.66	-0.28 ± 1.46	-0.38 ± 1.97	0.03 ± 1.62	0.03 ± 1.69	-0.20 ± 1.28	-0.20 ± 1.23	-0.35 ± 2.10	0.05 ± 1.66	-0.59 ± 1.70	-0.18 ± 1.03	0.747
y	0.12 ± 0.95	0.32 ± 1.57	0.23 ± 1.73	-0.17 ± 1.43	-0.44 ± 1.42	-0.04 ± 2.30	0.26 ± 0.85	0.13 ± 0.86	0.32 ± 1.61	-0.16 ± 0.96	0.39 ± 1.27	0.03 ± 1.14	0.545
z	-0.09 ± 0.71	-0.20 ± 1.23	0.46 ± 1.81	0.47 ± 2.00	0.39 ± 1.80	0.80 ± 2.26	0.04 ± 0.62	-0.18 ± 0.51	-0.17 ± 0.80	0.36 ± 0.69	-0.08 ± 0.54	0.21 ± 0.86	0.318

**Figure 3.**
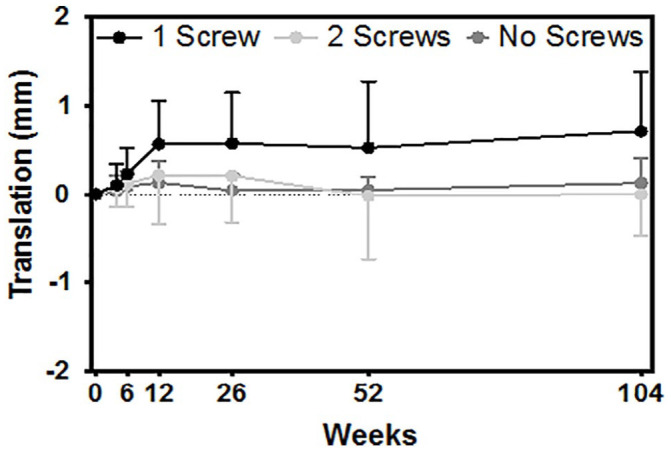
Comparing proximal migration (y-axis translation) between cups fixated with 1 or 2 screws (*p* = 0.013), 1 or no screws (*p* = 0.0004) and 2 or no screws (*p* = 0.758).

Within the screw fixation group, 24 patients received 1 screw and 18 patients received 2 screws. There was 1 patient who received 3 screws and was excluded from the following analysis. There was no difference in the inclination angle or the anteversion angle between these patients (*p* = 0.178, mean difference = 2.09° and *p* = 0.585, mean difference = 2.28°, respectively). The number of screws used to fixate the acetabulum cup had a significant impact on the proximal migration, with the cups fixated with 1 screw migrating significantly more than the cups fixated with 2 (Figure 3) (*p* = 0.013, mean difference = 0.712 mm). Compared to the group with no screw fixation, cups fixated with 1 screw migrated significantly more from 2 weeks to 2 years post operation (Figure 3) (*p* = 0.0004, mean difference = 0.582 mm). Contrarily, there was no difference in proximal migration between cups fixated with no screws and cups fixated with 2 screws (Figure 3) (*p* = 0.758, mean difference = 0.130 mm). Additionally, 3 patients who received only 1 screw had proximal migration greater than 1 mm, which is defined as unacceptable by Pijls et al.^7^

Further, there were 11 patients who were excluded from the presented analysis for deviating from their study arm; six patients underwent the DA approach but did not receive screws and five patients underwent the DL approach but received 2 screws. There was no significant difference in the inclination or the anteversion angle between these groups (*p* = 0.064, mean difference = 7.53° and *p* = 0.212, mean difference = 9.09°, respectively). Comparing these groups to one another, there was no significant impact of screw fixation on proximal migration of the acetabular cups (*p* = 0.152, mean difference = 0.549 mm).

## Discussion

THA remains one of the most important and common procedures of healthcare systems around the world and despite advancements in surgical techniques and implant designs, revision rates remain concerning. Aseptic loosening of the prosthetic components is a known risk factor for revision surgery and latest implant designs have incorporated mechanisms to increase initial stability, such as available screw holes in most acetabular prostheses. Still, the use of screws to supplement acetabular fixation is unsettled in literature, leaving the choice to surgeon preference.^24^ As such, the purpose of this study was to assess acetabular migration between cups fixated with and without the use of screws.

Initial cup position is an important determinant of THA success since cup malposition increases the risk of revision surgery, as it can lead to impingement, reduced range of motion, dislocation, wear and osteolysis, loosening and cup failure.^22,25^ The Lewinnick safe zone for cup orientation is commonly cited and suggests an orientation of 40° ± 10° for inclination and 15° ± 10° for anteversion.^19,20^ However, a research synthesis by Harrison et al.^26^ found recommended cup orientation in literature varied, ranging from 24° to 50° and 0° to 40° for inclination and anteversion, respectively. Our results showed a significant difference in both the inclination and anteversion angles between screw fixation and no screw fixation groups. The mean inclination angle, although significantly different between groups, both fall within the suggested safe range. Similarly, the mean anteversion angle of both patient groups falls within Lewinnick’s safe zone, with the screw fixation group having higher cup anteversion. A study by Chen et al.^27^ compared cup position between DA and DL THA approaches and found a tendency to antevert the cup with the DA approach, which could explain the slightly significant difference between patient groups. Ultimately, the position of the acetabular cup is left to surgeon preference, and each surgeon has their own targets for cup placement that are within the recommended safe zones. Further, although there was a difference in migration between cups fixated with 1 or 2 screws within the DA screw fixation group, there was no difference in the inclination and anteversion angles between these groups. This may suggest that cup position did not influence the migration of the cups or that the cup position chosen needs at least 2 fixation screws to minimise migration.

The results of this study revealed that the use of screws had a significant impact on the stability of the cup and resulted in increased proximal migration up to 2 years postoperatively. Previous RSA studies compared acetabular cup stability between press-fit cups fixated with no screws or with 2 or 3 screws and found no added benefit of screw fixation on cup stability.^28,29^ This is supported in our results, which also showed no difference in migration between cups fixated with no screws and cups fixated with 2 screws. There was also no difference when comparing migration of the excluded DL patients who received 2 screws and the excluded DA patients who received no screws, which may further support the finding between the use of no screws and 2 screws; although the sample size compared here is too small to make any conclusions. However, increased migration was detected in acetabular cups fixated with 1 screw, demonstrating that the number of screws is a contributing factor to increased stability. This is supported by Hsu et al.,^30^ who found increased torque stability and reduced micromotion between the use of 1 and 2 screws, concluding that 2 screws are recommended to better fixate the cup.^30,31^ It is possible that the insertion of only 1 screw may have interfered with the initial press-fit fixation, resulting in some micromotion, though below the threshold of unacceptable migration. Further, there were 3 patients in the screw fixation group who had proximal migration greater than 1 mm, which is defined as unacceptable by Pijls et al.^7^ The acetabular cups of all 3 of these patients were fixated with the use of only 1 screw, and the migration experienced could have potentially been mitigated with an additional screw.

Some limitations can be noted in the presented study. Primarily, this study was a secondary analysis and was not designed to detect differences in acetabular cup migration. Still, the power calculation analysis showed sufficient power to compare patient groups and results of this study are of important clinical significance. Additionally, potential bias may be introduced since there was a difference in the operative techniques used between screw fixation groups. However, an expertise-based design has been reported to reduce challenges to expertise bias and ensured the best technique was used in all cases, as both surgeons are experts in their respective approaches.^18^ Additionally, surgeon discretion of number of screws used for patients in the screw fixation group is a limitation of our study and highlights a need for more objective assessments of intraoperative stability. Further, although each surgeon has their own reaming technique, both underream by 1 mm to achieve their press-fit fixation of the acetabular cup. Another limitation is that there was no assessment made on patient bone quality, which could have had an impact on the stability of the cups. Additionally, the results of this study may not be generalisable to other acetabular cup designs as all patients received the same cementless Pinnacle cup.

In conclusion, this study demonstrated that the use of screws had an impact on the primary stability of the acetabulum cup. Specifically, cups fixated with 1 screw resulted in greater migration than compared to no screws or 2 screws, though the mean magnitude of migration was well below the 1.0-mm threshold for unacceptable migration. 3 patients who received only 1 screw did exceed the threshold for unacceptable migration. As such, the results of this study show that two screws to supplement cup fixation can provide good implant stability that is equivalent to a secure press-fit component with no screws.

## Supplemental Material

sj-pdf-1-hpi-10.1177_11207000231164711 – Supplemental material for Acetabular cup fixation with and without screws following primary total hip arthroplasty: migration evaluated by radiostereometric analysis Click here for additional data file. Supplemental material, sj-pdf-1-hpi-10.1177_11207000231164711 for Acetabular cup fixation with and without screws following primary total hip arthroplasty: migration evaluated by radiostereometric analysis by Jennifer S Polus, Edward M Vasarhelyi, Brent A Lanting and Matthew G Teeter in HIP International

## References

[1] Pabinger C Geissler A . Utilization rates of hip arthroplasty in OECD countries. Osteoarthritis Cartilage 2014; 22: 734–741. 24780823 10.1016/j.joca.2014.04.009

[2] Canadian Institute for Health Information. Hip and knee replacements in Canada, 2017–2018: Canadian Joint Replacement Registry Annual Report. Ottawa, ON: Canadian Institute for Health Information, 2019.

[3] Bozic KJ Kamath AF Ong K , et al. Comparative epidemiology of revision arthroplasty: failed THA poses greater clinical and economic burdens than failed TKA. Clin Orthop Relat Res 2015; 473: 2131–2138. 25467789 10.1007/s11999-014-4078-8 PMC4418985

[4] Kelmer G Stone AH Turcotte J , et al. Reasons for revision: primary total hip arthroplasty mechanisms of failure. J Am Acad Orthop Surg 2021; 29: 78–87. 32404682 10.5435/JAAOS-D-19-00860

[5] Long WJ Nayyar S Chen KK , et al. Early aseptic loosening of the Tritanium primary acetabular component with screw fixation. Arthroplast Today 2018; 4: 169–174. 29896547 10.1016/j.artd.2017.11.009 PMC5994600

[6] Klerken T Mohaddes M Nemes S , et al. High early migration of the revised acetabular component is a predictor of late cup loosening: 312 cup revisions followed with radiostereometric analysis for 2-20 years. Hip Int 2015; 25: 471–476. 25952912 10.5301/hipint.5000246

[7] Pijls BG Nieuwenhuijse MJ Fiocco M , et al. Early proximal migration of cups is associated with late revision in THA: a systematic review and meta-analysis of 26 RSA studies and 49 survival studies. Acta Orthop 2012; 83: 583–591. 23126575 10.3109/17453674.2012.745353 PMC3555453

[8] Hsu JT Chang CH An KN , et al. Effects of screw eccentricity on the initial stability of the acetabular cup. Int Orthop 2007; 31: 451–455. 16947050 10.1007/s00264-006-0226-4 PMC2267626

[9] Brodt S Bischoff K Schulze M , et al. The use of acetabular screws in total hip arthroplasty and its influence on wear and periacetabular osteolysis in the long-term follow-up. Int Orthop 2022; 46: 717–722. 34581866 10.1007/s00264-021-05219-7 PMC8930858

[10] García-Rey E García-Cimbrelo E Cruz-Pardos A . Cup press fit in uncemented THA depends on sex, acetabular shape, and surgical technique. Clin Orthop Relat Res 2012; 470: 3014–3023. 22576930 10.1007/s11999-012-2381-9 PMC3462870

[11] Fei C Wang PF Wei W , et al. Relationship between use of screws and acetabular cup stability in total hip arthroplasty: a meta-analysis. J Int Med Res 2020; 48: 1–11. 10.1177/0300060520903649 PMC7111112 32054354

[12] Tabata T Kaku N Hara K , et al. Initial stability of cementless acetabular cups: press-fit and screw fixation interaction—an in vitro biomechanical study. Eur J Orthop Surg Traumatol 2015; 25: 497–502. 25421640 10.1007/s00590-014-1571-4 PMC4363363

[13] Nugent M Campbell DG Lewis PL , et al. Acetabular screws do not improve early revision rates in primary total hip arthroplasty. An instrumented registry analysis. Int Orthop 2021; 45: 593–604. 33479835 10.1007/s00264-021-04949-y

[14] Campbell D Mercer G Nilsson KG , et al. Early migration characteristics of a hydroxyapatite-coated femoral stem: an RSA study. Int Orthop 2011; 35: 483–488. 20012862 10.1007/s00264-009-0913-z PMC3066322

[15] Karrholm J Gill RHS Valstar ER . The history and future of radiostereometric analysis. Clin Orthop Relat Res 2006; 448: 10–21. 16826090 10.1097/01.blo.0000224001.95141.fe

[16] Polus JS Perelgut ME Vasarhelyi EM , et al. Femoral stem migration after direct lateral and direct anterior total hip arthroplasty: a prospective cohort study. Can J Surg 2022; 65: E487–E495. 10.1503/cjs.013221 PMC9363127 35926882

[17] Mundi R Chaudhry H Mundi S , et al. Design and execution of clinical trials in orthopaedic surgery. Bone Joint Res 2014; 3: 161–168. 24869465 10.1302/2046-3758.35.2000280 PMC4097861

[18] Devereaux PJ Bhandari M Clarke M , et al. Need for expertise based randomised controlled trials. BMJ 2005; 330: 88–91. 15637373 10.1136/bmj.330.7482.88 PMC543877

[19] Lewinnek GE Lewis JL Tarr R , et al. Dislocations after total hip-replacement arthroplasties. J Bone Joint Surg Am 1978; 60: 217–220. 641088

[20] Murphy WS Yun HH Hayden B , et al. The safe zone range for cup anteversion is narrower than for inclination in THA. Clin Orthop Relat Res 2018; 476: 325–335. 29529664 10.1007/s11999.0000000000000051 PMC6259696

[21] Mechlenburg I . Evaluation of Bernese periacetabular osteotomy. Acta Orthop 2008; 79: 1–43. 18853289 10.1080/17453690610046558

[22] Tiberi JV Pulos N Kertzner M , et al. A more reliable method to assess acetabular component position. Clin Orthop Relat Res 2012; 470: 471–476. 21822569 10.1007/s11999-011-2006-8 PMC3254767

[23] Scholarship@Western and Goyal P . Effects of acetabular positioning in total hip arthroplasty, https://ir.lib.uwo.ca/etd (2015, accessed 25 January 2022).

[24] Ni S Luo P Guo L , et al. Are additional screws required for press-fit fixation of cementless acetabular cups? A systematic review and meta-analysis. J Orthop Traumatol 2022; 23: 1–14. 35142933 10.1186/s10195-022-00629-8 PMC8831679

[25] Bhaskar D Rajpura A Board T . Current concepts in acetabular positioning in total hip arthroplasty. Indian J Orthop 2017; 51: 386–396. 28790467 10.4103/ortho.IJOrtho_144_17 PMC5525519

[26] Harrison CL Thomson AI Cutts S , et al. Research synthesis of recommended acetabular cup orientations for total hip arthroplasty. J Arthroplasty 2014; 29: 377–382. 23958234 10.1016/j.arth.2013.06.026

[27] Chen AF Chen CL Low S , et al. Higher acetabular anteversion in direct anterior total hip arthroplasty: a retrospective case-control study. HSS J 2016; 12: 240–244. 27703417 10.1007/s11420-016-9488-6 PMC5026652

[28] Minten MJM Heesterbeek PJC Spruit M . No effect of additional screw fixation of a cementless, all-polyethylene press-fit socket on migration, wear, and clinical outcome. Acta Orthop 2016; 87: 363–367. 27299418 10.1080/17453674.2016.1190244 PMC4967278

[29] Otten VTC Crnalic S Röhrl SM , et al. Stability of uncemented cups — long-term effect of screws, pegs and HA coating: a 14-year RSA follow-up of total hip arthroplasty. J Arthroplasty 2016; 31: 156–161. 10.1016/j.arth.2015.07.012 26260783

[30] Hsu JT Lai KA Chen Q , et al. The relation between micromotion and screw fixation in acetabular cup. Comput Methods Programs Biomed 2006; 84: 34–41. 16971018 10.1016/j.cmpb.2006.08.002

[31] Hsu JT Chang CH Huang HL , et al. The number of screws, bone quality, and friction coefficient affect acetabular cup stability. Med Eng Phys 2007; 29: 1089–1095. 17194616 10.1016/j.medengphy.2006.11.005

