# Supplementary material for: Acetabular cup fixation with and without screws following primary total hip arthroplasty: migration evaluated by radiostereometric analysis
Source: Hip Int. 2023 Apr 5;34(1):42–8. doi: 10.1177/11207000231164711 (PMC10787387; doi:10.1177/11207000231164711)
Supplement: sj-pdf-1-hpi-10.1177_11207000231164711 – Supplemental material for Acetabular cup fixation with and without screws following primary total hip arthroplasty: migration evaluated by radiostereometric analysis [file sj-pdf-1-hpi-10.1177_11207000231164711.pdf]

Supplementary Materials

Supplemental Table 1: Questionnaires between patient groups reported as mean ± standard deviation.

Questionnaire	Screw Fixation	No Screw Fixation	P-Value
SF-12 Mental			
Pre-Operation	55.03 ± 8.30	55.75 ± 10.06	0.745
3-Month Postoperation	58.25 ± 5.66	57.94 ± 6.69	0.849
1-Year Postoperation	57.14 ± 5.59	56.20 ± 8.53	0.613
2-Years Postoperation	53.24 ± 17.64	50.93 ± 19.50	0.685
SF-12 Physical			
Pre-Operation	34.36 ± 10.10	34.48 ± 10.21	0.962
3-Month Postoperation	48 15 ± 9.18	47.01 ± 8.12	0.631
1-Year Postoperation	51.79 ± 8.38	51.94 ± 7.85	0.948
2-Years Postoperation	48.58 ± 16.45	45.35 ± 18.09	0.544
WOMAC Pain			
Pre-Operation	52.79 ± 16.56	49.38 ± 13.93	0.396
3-Month Postoperation	85.38 ± 26.97	85.18 ± 19.34	0.976
1-Year Postoperation	91.62 ± 16.63	97.38 ± 4.36	0.126
2-Years Postoperation	95.88 ± 7.54	94.33 ± 12.80	0.598
WOMAC Function			
Pre-Operation	49.45 ± 18.24	51.47 ± 16.65	0.656
3-Month Postoperation	86.80 ± 14.02	87.05 ± 9.47	0.943
1-Year Postoperation	92.45 ± 16.54	94.12 ± 9.20	0.672
2-Years Postoperation	94.98 ± 7.55	92.06 ± 11.28	0.291
WOMAC Total			
Pre-Operation	50.60 ± 15.64	48.42 ± 13.74	0.570
3-Month Postoperation	85.42 ± 12.44	85.06 ± 9.58	0.908
1-Year Postoperation	90.64 ± 17.17	94.10 ± 8.36	0.390
2-Years Postoperation	93.73 ± 8.27	91.71 ± 11.92	0.497
UCLA			
Pre-Operation	5.53 ± 1.65	5.64 ± 2.25	0.826
3-Month Postoperation	6.44 ± 1.50	6.42 ± 1.41	0.953
1-Year Postoperation	7.13 ± 1.49	7.27 ± 1.80	0.730
2-Years Postoperation	7.14 ± 1.51	7.36 ± 1.71	0.595
HHS Total			
Pre-Operation	57.74 ± 10.89	59.82 ± 9.57	0.453
3-Month Postoperation	97.59 ± 3.34	96.00 ± 5.92	0.359
1-Year Postoperation	96.88 ± 6.67	98.33 ± 3.04	0.535
2-Years Postoperation	97.50 ± 4.40	95.40 ± 4.93	0.493

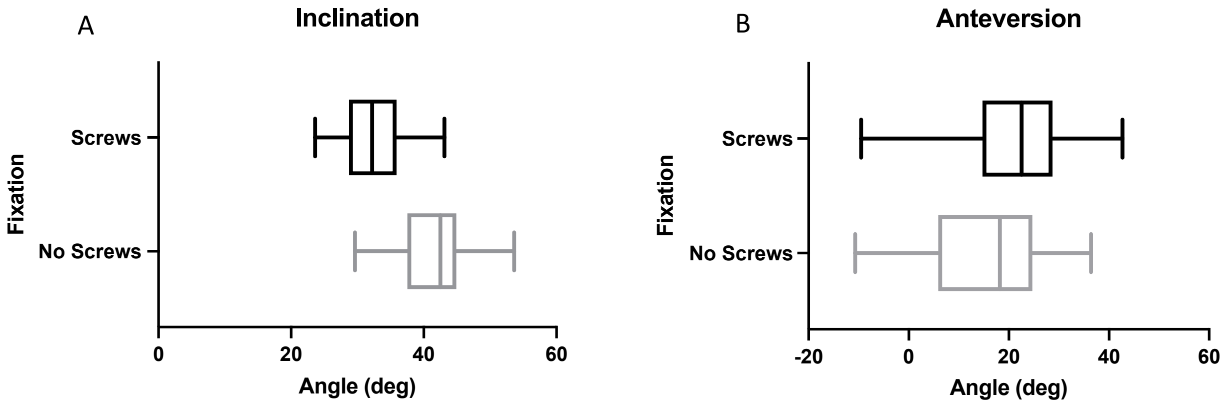

Supplemental Figure 1: Comparing radiographic acetabular cup position at six-weeks post-operation between patient groups: A) inclination ($p<0.0001$) and B) anteversion ($p=0.0499$).
